# Integrating Lipidomics and Transcriptomics Reveals the Crosstalk Between Oxidative Stress and Neuroinflammation in Central Nervous System Demyelination

**DOI:** 10.3389/fnagi.2022.870957

**Published:** 2022-04-25

**Authors:** Zhi-jie Zhao, Rui-zhe Zheng, Xiao-jing Wang, Tong-qi Li, Xiao-hua Dong, Chang-yi Zhao, Xin-yuan Li

**Affiliations:** ^1^Department of Neurosurgery, Tongren Hospital, Shanghai Jiao Tong University School of Medicine, Shanghai, China; ^2^Department of Neurosurgery, Huashan Hospital, Shanghai Medical College, Fudan University, Shanghai, China; ^3^Department of Neurology, The First Affiliated Hospital of Anhui Medical University, Hefei, China; ^4^Department of Ophthalmology, Shanghai General Hospital, Shanghai Jiao Tong University School of Medicine, Shanghai, China; ^5^Hongqiao International Institute of Medicine, Tongren Hospital, Shanghai Jiao Tong University School of Medicine, Shanghai, China

**Keywords:** targeted lipidomics, oxidative stress, mitochondrial dysfunction, neuroinflammation, crosstalk

## Abstract

Multiple sclerosis (MS) is an incurable and progressive neurodegenerative disease that affects more than 2.5 million people worldwide and brings tremendous economic pressures to society. However, the pathophysiology of MS is still not fully elucidated, and there is no effective treatment. Demyelination is thought to be the primary pathophysiological alteration in MS, and our previous study found abnormal lipid metabolism in the demyelinated corpus callosum. Growing evidence indicates that central nervous system (CNS) demyelinating diseases never result from one independent factor, and the simultaneous participation of abnormal lipid metabolism, oxidative stress, and neuroinflammation could potentiate each other in the pathogenesis of MS. Therefore, a single omics analysis cannot provide a full description of any neurodegenerative disease. It has been demonstrated that oxidative stress and neuroinflammation are two reciprocal causative reasons for the progression of MS disease. However, the potential crosstalk between oxidative stress and neuroinflammation remains elusive so far. With an integrated analysis of targeted lipidomics and transcriptomics, our research presents the potential interaction between abnormalities of lipid metabolism, mitochondrial dysfunction, oxidative stress, and neuroinflammation in CNS demyelinating diseases. The findings of this paper may be used to identify possible targets for the therapy of CNS demyelinating diseases.

## Introduction

Central nervous system (CNS) demyelinating diseases are characterized by inflammatory stimulation, vascular compression, immune abnormalities, and massive loss of oligodendrocytes (Sen et al., 2019). The destruction of the myelin sheath will lead to neurological disorders, including multiple sclerosis (MS) and cranial nerve disorders (Kim et al., 2021; Saraswat et al., 2021). MS is a progressive, incurable disease that affects more than 2.5 million individuals globally (Zhao et al., 2020). Patients with MS have a variety of neurological symptoms such as motor impairment, cognitive impairment, depressive tendencies, visual impairment, sleep disturbances, and anxiety tendencies, all of which negatively affect the quality of life and lead to a significant impact on medical and healthcare systems (Shao et al., 2021; Zhao et al., 2021). However, to date, the comprehensive pathogenesis of MS remains unknown, and there is no effective therapy (Meng-Ru et al., 2021).

The pathogenic processes of MS are astoundingly complicated. Previous research has suggested that mitochondrial dysfunction (Luo et al., 2020), oxidative stress (Shiri et al., 2020; Mihai et al., 2021), and neuroinflammation (Meng-Ru et al., 2021; Shao et al., 2021) may be jointly involved in the onset and progression of MS. Cellular energy crisis and increased oxidative stress are consequences of mitochondrial dysfunction. Defective mitochondrial enzyme activity plays a critical role in the development and progression of MS (Lu et al., 2000). Additionally, mitochondrial malfunction and an increase in the formation of reactive oxygen species (ROS) can cause oligodendrocyte destruction (Yeung et al., 2021). Moreover, ROS can interact with biological substances such as DNA or lipids (Shiri et al., 2020). Thus, excessive ROS production can trigger mitochondrial dysfunction, which manifests itself through aberrant gene expression, enzyme activity, and kinetics in the mitochondria (Mao and Reddy, 2010). In addition, it is thought that pro-inflammatory conditions in the CNS accelerate myelin sheath degradation (Shao et al., 2021).

Metabolomics widens our knowledge of small molecule metabolites, which are often considered as biomarkers, end products of cellular metabolism, while their regulatory roles in the genome, transcriptome, and proteome are often overlooked. Our prior investigation discovered an abnormal lipid metabolism in demyelinated central nerve tissue (Zhao et al., 2021). At present, there is no obvious association between abnormal lipid metabolism and mitochondrial dysfunction, oxidative stress, and neuroinflammation in CNS demyelinated tissues. We employed combined targeted lipid metabolomics and transcriptomics to investigate the potential interaction between them in demyelinating tissue of the central nervous system.

## Materials and Methods

### Animals and Experimental Design

Male C57BL/6 mice (8 weeks old) were purchased from Spelford (Beijing) Biotechnology Company. Mice were housed in an air-conditioned room at 22 ± 1^°^C with a 12-h light-dark cycle (lights on from 7:00 a.m. to 7:00 p.m.) (Zhao et al., 2021). Food and tap water were freely available. All procedures for the following experiments were approved by the Animal Care and Use Committee, performed by the NIH Guide for the Care and Use of Laboratory Animals.

Twenty male C57BL/6 mice (8 weeks old) were randomly divided into the control group (*n* = 10) and the cuprizone (CPZ) group (*n* = 10), and mice in the CPZ group were fed by a diet containing 0.2% cuprizone (reagent purchased from Sigma, custom-made feed from Jiangsu Hershey Feeds). By referring to the literature (Wellman et al., 2020), C57BL/6 male mice were selected to be fed 0.2% cuprizone chow to construct the demyelination model, and we chose continuous feeding (11 weeks) for the CPZ group (Zhao et al., 2021). Then, mice were executed, and the corpus callosum was immediately stored at −80°C for subsequent detection of the targeted lipidomics and transcriptomics.

### Corpus Callosum Lipid Extraction

The Corpus Callosum was thawed on ice. Take 20 mg of sample and homogenize with 1 ml of the mixture (including methanol, MTBE, and internal standard mixture) and a steel ball. Remove the pellet and vortex the mixture for 15 min. Add 200 μL of water, vortex for 1 min, and then centrifuge at 4°C for 10 min at 12,000 rpm. Remove 300 μL of supernatant and concentrate. Dissolve the powder with 200 μL of the reagent solution and store it at −80°C. Place the solution in a sample vial for LC-MS/MS analysis. In addition, to investigate the stability of instruments and the reproducibility of the sample, quality control (QC) samples were also prepared by combining equal volumes of each sample.

### Acquisition of Targeted Lipidomics Data

HPLC grades of acetonitrile (ACN), methanol (MeOH), isopropanol (IPA), methylene chloride (CH2Cl2), and tert-butyl methyl ether (MTBE) were purchased from Merck (Darmstadt, Germany). HPLC grade formic acid (FA) and ammonium formate (AmFA) were purchased from Sigma-Aldrich (St. Louis, MO, United States). Ultrapure water was obtained using a Milli-Q system (Millipore, Billerica, MA). Lipid standards were purchased from Sigma-Aldrich or Avanti Polar Lipids (Alabaster, AL).

Sample extracts were analyzed using an LC-ESI-MS/MS system (UPLC, ExionLC AD,^1^ MS, QTRAP^®^ 6500+ system^2^). The analytical conditions were as follows, UPLC: column, Thermo Accucore™ C30 (2.6 μm, 2.1 mm × 100 mm i.d.); solvent system, A: acetonitrile/water (60/40, V/V, 0.1% formic acid, 10 mmol/L ammonium formate), B: acetonitrile/isopropanol (10/90 VV/V, 0.1% formic acid, 10 mmol) /L ammonium formate); gradient program, A/B (80:20, V/V) at 0 min, 70:30 V/V, 2.0 min, 40:60 V/V, 4 min, 15:85 V/V, 9 min, 10 :90 V/V at 14 min, 5:95 V/V at 15.5 min, 5:95 V/V at 17.3 min, 80:20 V/V V at 17.3 min, 80:20 V/V at 20 min; flow rate, 0.35 mL/min; temperature, 45°C; injection volume: 2 μL. Alternatively, connect the discharge port to an ESI triple quadrupole linear ion trap (QTRAP)-MS.

LIT and triple quadrupole scanning (QQQ) are performed using a triple quadrupole linear ion trap mass spectrometer (QTRAP). The QTRAP^®^ 6500+ LC-MS/MS system is equipped with an ESI turbo ion spray interface operating in positive ion mode and negative ion mode, controlled by Analyst 1.6.3 (Sciex) software. The operating parameters of the ESI source are as follows: ion source, turbo spray; source temperature 500°C; ion spray voltage (IS) 5,500 V (positive), −4,500 V (negative); ion source gas 1 (GS1), gas 2 (GS2), and gas curtain gas (CUR) set to 45, 55, and 35 psi, respectively. QQQ and LIT modes used 10 and 100 μ mol/L polypropylene glycol solution for device tuning and mass calibration in QQQ and LIT modes. QQQ scans were captured as MRM experiments with the collision gas (nitrogen) set to 5 psi. Individual MRM conversions of DP and CE were done by further DP and CE optimization. A specific set of MRM conversions was monitored for each period based on the metabolites eluted during that period.

### RNA Sequencing

The process of RNA sequencing includes RNA extraction, RNA detection, library construction, and up-sequencing. Briefly, RNA degradation and contamination were monitored on 1% agarose gels, followed by RNA purity. RNA purity was checked using the NanoPhotometer^®^ spectrophotometer (IMPLEN, CA, United States). Then, the RNA concentration was measured using Qubit^®^ RNA Assay Kit in Qubit^®^2.0 Flurometer (Life Technologies, CA, United States). After that, RNA integrity was assessed using the RNA Nano 6000 Assay Kit of the Bioanalyzer 2100 system (Agilent Technologies, CA, United States). A total amount of 1 μg RNA per sample was used as input material for the RNA sample preparations. Following the manufacturer’s recommendations, the sequencing libraries were generated using NEBNext^®^ Ultra™ RNA Library Prep Kit for Illumina^®^ (NEB, United States), and index codes were added to attribute sequences to each sample.

Purify mRNA from total RNA using poly-T oligo-binding magnetic beads. High-temperature lysis in NEBNext First Strand Synthesis Reaction Buffer (5X) using divalent cations. The first strand of cDNA was synthesized using random hexamer primers and M-MuLV reverse transcriptase (RNase H-). The second strand of the cDNA is then synthesized using DNA polymerase I and RNase H. The remaining prominent ends are converted to flat ends by exonuclease/polymerase activity. After adenylation of the 3′ end of the DNA fragment, the NEBNext splice is ligated to the hairpin loop structure and prepared for hybridization. To preferably select cDNA fragments of 250–300 bp in length, library fragments were purified using the AMPure XP system (Beckman Coulter, Beverly, United States). Then 3 μl USER enzyme (NEB, United States) was used with the size-selected, aptamer-linked cDNA for 15 min at 37°C followed by 5 min at 95°C before PCR. PCR was then performed using Phusion high-fidelity DNA polymerase, universal PCR primers, and index (X) primers. Finally, the PCR products were purified (AMPure XP system), and the quality of the library was assessed on an Agilent Bioanalyzer 2100 system. According to the manufacturer’s instructions, index-coded samples were clustered on the cBot cluster generation system using the TruSeq PE Cluster Kit v3-cBot-HS (Illumia). After cluster generation, library preparations were sequenced on the Illumina Hiseq platform, and 125/150 bp double-end reads were generated.

### Statistical Analyses

#### Data Analysis of Lipidomics

Unsupervised principal component analysis (PCA) was performed by R software.^3^ The data were scaled with unit variance prior to unsupervised PCA. The results of HCA (hierarchical cluster analysis) for samples and metabolites are shown as heat maps with tree plots etc., while the Pearson correlation coefficients (PCC) between samples were calculated using the cor function in R software and are shown as heat maps. The normalized signal intensity (scale per unit variance) of metabolites was shown as a chromatogram for HCA. Metabolites that were significantly regulated between groups were identified by VIP ≥ 1, fold change ≥ 1.5 or fold change ≤ 0.66, *p*-value < 0.05. VIP values were extracted from the OPLS-DA results, containing score and alignment plots, and were created using the R package MetaboAnalystR. Before OPLS-DA, the data were log-transformed (log_2_) and centered on the mean. Permutation tests (200 permutations) were performed to avoid over-fitting. The identified metabolites were annotated with the help of the kyoto encyclopedia of genes and genomes (KEGG) Compound Database,^4^ and the annotated metabolites were then added to the KEGG Pathway Database.^5^ Pathways with significantly regulated metabolites were then fed into Metabolite Set Enrichment Analysis (MSEA); in addition, their significance was determined by the *p*-value of the hypergeometric test’s *p*-values.

#### Data Analysis of Transcriptomics

Raw data is filtered with fastp (V 0.19.3), mainly by removing reads with adapter; paired reads are removed when sequencing reads contain more than 10% of the number of bases in the read; if a sequencing read contains more than 50% of the number of bases in a low quality read (Q ≤ 20), the paired read is removed. All subsequent analyses are based on clear reads. Download the reference genome and its annotation file from the website, build the index using HISAT (V2.1.0) and compare the clean reads to the reference genome. The StringTie (V1.3.4d) was used to predict new genes. StringTie applies a network streaming algorithm and optionally splices transcripts from scratch. Compared to the soft of Cufflinks, StringTie can create more complete and accurate transcripts; in addition, it is faster than Cufflinks. The featureCounts (V1.6.2) was used to calculate Gene alignment, and then the FPKM was used to calculate each gene based on gene length. DESeq2 (V1.22.1) was used to analyze the differential expression between the two groups and corrected for *P*-values using Benjamini & Hochberg’s method. The corrected *P*-value and | log_2_foldchange | were used as thresholds for expressing significant differences. The enrichment analysis is performed based on hypergeometric tests. In KEGG, the hypergeometric distribution test is performed in terms of paths; For GO, it is performed in GO terms. The analysis of protein interactions for the differentially expressed genes is based on the STRING database^6^ with known and predicted protein-protein interactions. We constructed the network for species present in the database by extracting a list of target genes from the database. Otherwise, we used Blast (v2.7.1+) to compare the target gene sequences with selected reference protein sequences and then constructed the network based on known interactions for the selected reference species.

#### Integrated Analysis of Targeted Lipidomics and Transcriptomics

Pearson correlation analysis was used to determine the correlation coefficients between differential metabolites and genes. In addition, we selected the differential metabolites and genes with a correlation coefficient >0.6 to plot the map of the correlation network using the Cytoscape software (v3.8.2).

## Results

### Data Pre-processing and Quality Control of Lipidomics

**FIGURE 1:**
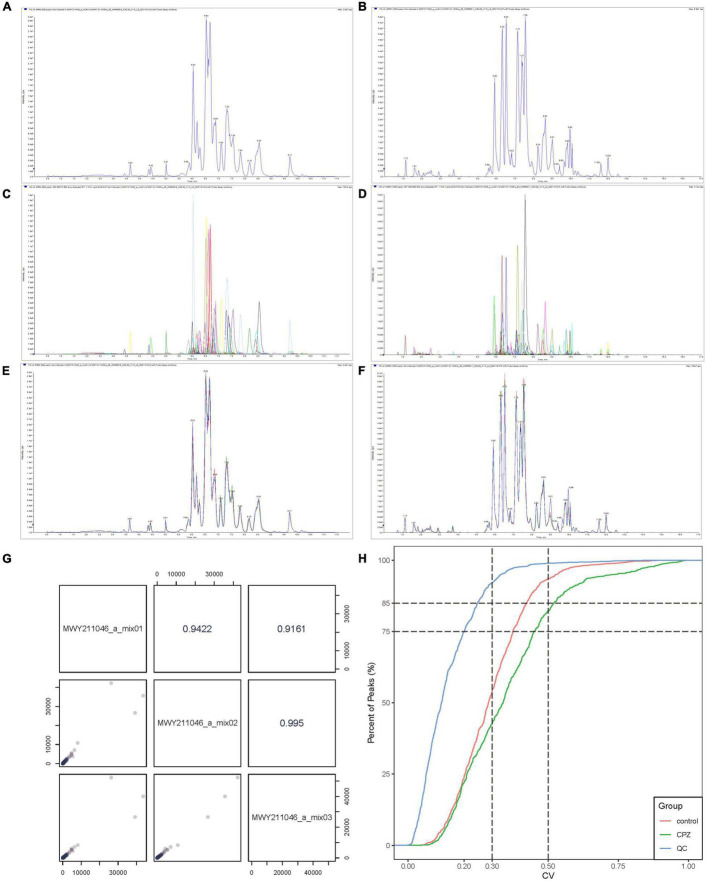
**(A)** The TIC of mixed QC sample in negative mode. **(B)** The TIC of mixed QC sample in positive mode. **(C)** The MRM of mixed QC sample in negative mode. **(D)** The MRM of mixed QC sample positive mode. **(E)** The overlapping curve of TIC in negative mode. **(F)** The overlapping curve of TIC in positive mode. **(G)** The correlation analysis diagram of QC samples. **(H)** The CV diagram of QC samples.

The Software Analyst (v1.6.3) was used to process mass spectrometry data. The intensity of all ions in the mass spectra at each time point of the mixed QC sample was summed and scanned continuously to obtain the total ions current (TIC) (Figure 1A: Negative mode; Figure 1B: Positive mode), the multi-matter extracted ion flow spectroscopy was used to draw multi-peak maps of MRM metabolite detection (Figure 1C: Negative mode; Figure 1D: Positive mode). The horizontal coordinate is the retention time for metabolite detection, and the vertical coordinate is the ion flow intensity. According to the lipid database, the detected lipid metabolites were characterized by mass spectrometry. The mass spectral peaks detected for each substance in the different samples were corrected to ensure accurate quantification.

The instrument’s stability was evaluated by overlapping TIC (Figure 1E: Negative mode; Figure 1F: Positive mode) of the same QC sample. The results showed a high-level overlap of the TIC curves for metabolite detection, suggesting that the signal stability of the mass spectrometer was relatively stable when the same sample was detected at different times, and the signal was steady throughout the analysis processing. The Pearson correlation analysis was performed on the QC samples; the results showed that the testing process is relatively stable (Figure 1G). The coefficient of Variation (CV) can reflect the degree of data dispersion. The results of CV showed that in QC samples, the ratio of substances’ CV value less than 0.5 was higher than 85%, and the percentage less than 0.3 was higher than 75%, indicating that the experimental data were very stable (Figure 1H).

### Overall Samples Principal Component Analysis and Cluster Analysis of Lipidomics

**FIGURE 2:**
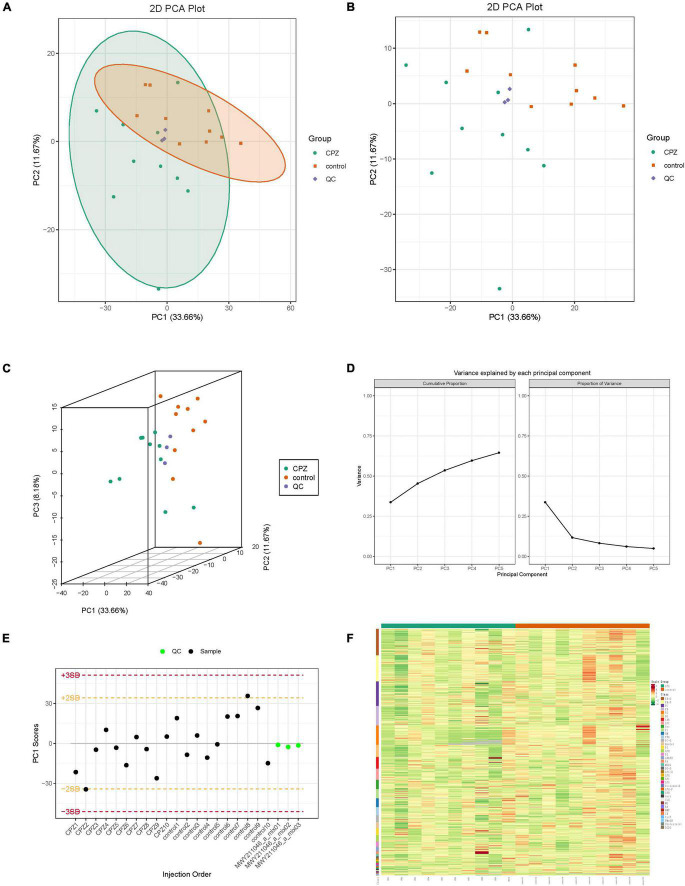
**(A,B)** The two-dimensional images of the overall samples PCA results. **(C)** The 3D images of the overall samples PCA results. **(D)** The plot of variance explained by each principal component. **(E)** The plot of principal component univariate statistical process control, the horizontal coordinates of the graph are the order of sample testing, the vertical coordinates reflect the values of PC1, and the yellow and red lines define the plus or minus 2 and 3 standard deviation ranges, respectively. The green dots represent QC samples, and the black dots represent test samples. **(F)** Cluster Analysis of Lipidomics.

The overall samples (includes QC samples) PCA analysis showed a trend of separation for lipid metabolites between the CPZ and control groups (Figures 2A–D). The horizontal coordinates of the PCA analysis plot indicate the first principal component, the vertical coordinates indicate the second principal component, and the percentage indicates the contribution of the principal component to the sample variation. Each point in the plot indicates a sample. The plot of principal component univariate statistical process control showed the PC1 of QC samples within plus or minus 3 standard deviations (SD); it indicated that the condition of the instrument is stable (Figure 2E). The content of lipid metabolites was normalized using the polar difference method. After normalization, the Hierarchical Cluster Analysis (HCA) was performed by R software (see text footnote 3) to analyze the accumulation pattern of metabolites among different samples (Figure 2F).

### Lipid Metabolites Composition and Changes in Subclass Content Between Cuprizone Group and Control Group

**FIGURE 3:**
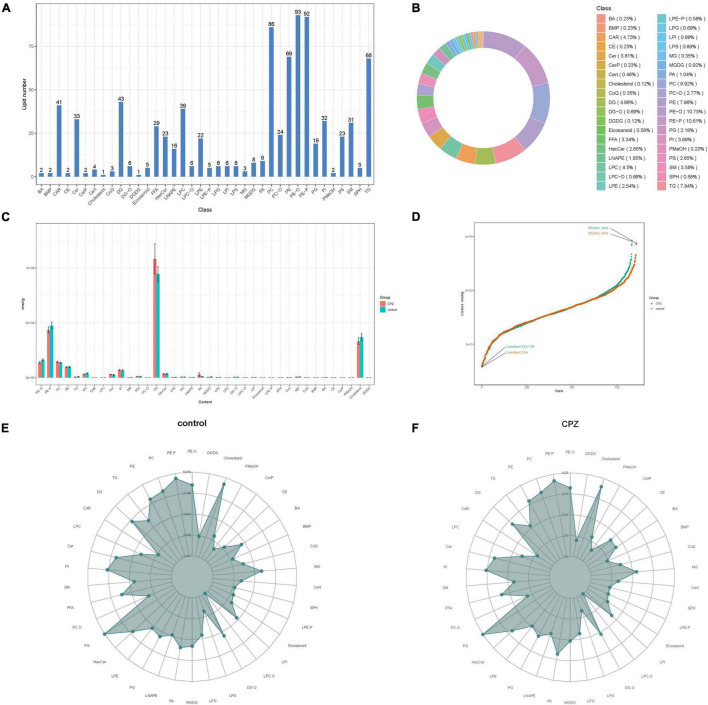
**(A)** The histogram of lipid subclass. **(B)** The ring diagram of lipid subclass composition (each color represents a lipid subclass, and the area of the color block indicates the proportion of that subclass). **(C)** The histogram of changes in lipid subclass content between CPZ and control groups. **(D)** The plot of dynamic distribution for lipid content (Each point in the graph represents a lipid molecule. The vertical coordinates represent the content corresponding to each lipid molecule, and the lipid molecules with the lowest and highest content are marked. Different color curves represent different groupings). **(E,F)** The radar charts of the content of the lipid metabolites between the CPZ and control groups (The grid lines indicate the LG categorical content, and the green shading consists of the line connecting each categorical content).

The statistics of the detected lipid subclasses, proportions of lipid subclasses, and the number of lipid metabolites contained in each sub-classes were shown in Figures 3A,B. The changes in lipid subclass content between the CPZ and the control group was shown in Figure 3C. The dynamic distribution of lipid content demonstrated the lowest and highest lipid metabolites in the CPZ and control groups, as well as the variation in lipid content across the range (Figure 3D). The radar charts showed the trend changes in the content of the lipid metabolites between the CPZ and control groups (Figures 3E,F).

### Subgroup Principal Component Analysis and Orthogonal Partial Least Squares Discriminant Analysis of Lipid Metabolites Between Cuprizone and Control Groups

**FIGURE 4:**
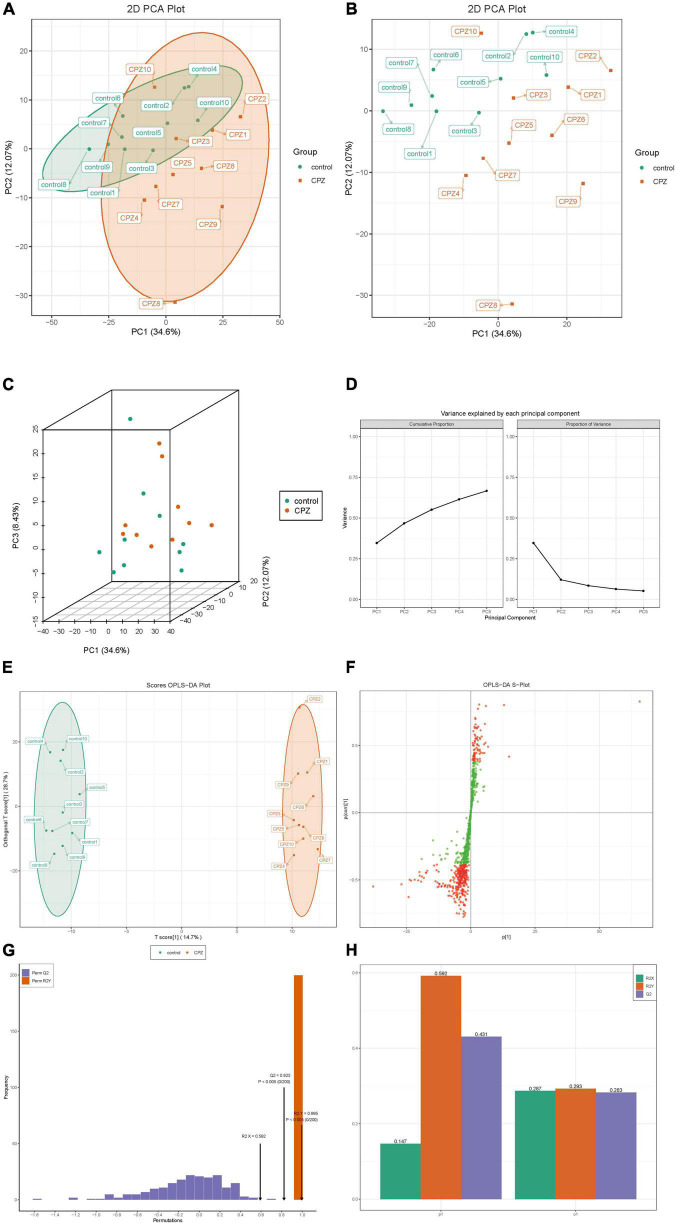
**(A,B)** The two-dimensional images of the subgroup PCA analysis. **(C)** The 3D images of the subgroup PCA analysis. **(D)** The plot of variance explained by each principal component. **(E)** OPLS-DA score map. **(F)** S-plot of OPLS-DA. **(G)** Permutation test. **(H)** The details of the permutation test.

The plots of subgroup PCA analysis were shown in Figure 4. The ellipse in Figure 4A is the confidence intervals of PCA plots, where the horizontal coordinate is the first principal component (PC1), and the vertical coordinate is the second principal component (PC2). According to the two-dimensional (Figures 4A,B) and three-dimensional PCA plots (Figure 4C), there was a significant trend of separation between the CPZ and control groups, which indicated a significant difference in lipid metabolites between the CPZ and control groups. The explainable variances of the Top five principal components are shown in Figure 4D. The horizontal coordinates indicate the individual principal components, and the vertical coordinates indicate the explainable variances; in addition, the left panel shows the cumulative explainable variances, and the right panel shows the explainable variances of each principal component.

The raw lipidomic data were presented using an OPLS-DA score plot, indicating a clear distinction between CPZ and control groups (Figure 4E). The subsequent permutation test of the generated model showed R2X = 0.592, R2Y = 0.995, Q2 = 0.823 (Figures 4G), confirming that this model performed reliably in terms of predictive performance. Figure 4F shows the S-plot of OPLS-DA in which the metabolites near the upper right and lower left corners indicate differential expression (red dots, VIP ≥ 1; green dots, VIP < 1). Figure 4H shows the details of the permutation test of the OPLS-DA model, including the values of R2X, Q2, and R2Y in orthogonal principal components (o1) and prediction of principal components(p1).

### Identification of Lipid Metabolites

**FIGURE 5:**
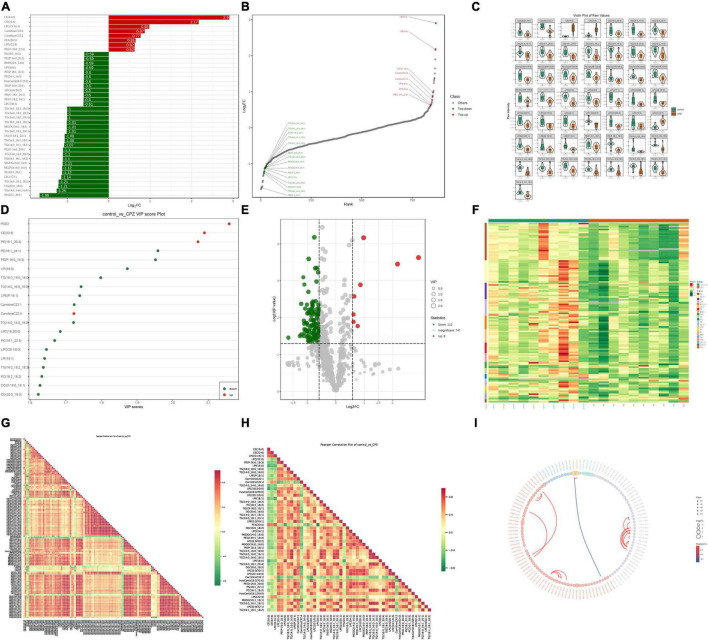
**(A)** Metabolite bar graph. **(B)** Dynamic distribution of metabolite content differences. **(C)** Metabolite violin diagram. **(D)** Metabolite VIP value chart. **(E)** Volcano Plot of metabolites. **(F)** Metabolite clustering heat map. **(G)** Heat map of the correlation of the all-differential metabolites. **(H)** Heat map of the correlation of the top 50 differential metabolites. **(I)** Differential metabolite chord diagram.

A total of 867 lipid metabolites were identified in this study, and 120 significantly different lipid metabolites (fold change ≥ 1.5 or fold change ≤ 0.66; VIP ≥ 1; *p*-value < 0.05) were screened. Compared to the control group, 8 lipid metabolites were up-regulated, and 112 were down-regulated in the CPZ group. A metabolite bar graph was created based on the magnitude of the Log_2_FC values for lipid metabolites (Figure 5A). To show the overall differences of metabolite more clearly and visually, the dynamic distribution of metabolite’ content difference was plotted based on FC values (Figure 5B). Based on the VIP values of the metabolites, a violin plot of the top 50 lipid metabolites was created to show the data distribution and its probability density (Figure 5C). The top 20 differential metabolites with the most significant VIP values in the OPLS-DA model were selected to plot the graph of VIP values (Figure 5D). Volcano Plot was used to demonstrate the differences in metabolite content between the two groups of samples and the statistical significance of the differences (Figure 5E). To facilitate the observation of the variation pattern of metabolite content, we applied normalization (Unit Variance Scaling, UV Scaling) to the raw content of the differential metabolites by row. We plotted the clustering heat map (Figure 5F). We used Pearson correlation analysis to determine the correlation of the differential metabolites and created a heat map of the correlation for the differential metabolites (Figures 5G,H). The differential metabolite chord diagram is shown in Figure 5I, and the plots were made by default for differential metabolites with |*r*| > 0.8 and *P* < 0.05.

### Kyoto Encyclopedia of Genes and Genomes Pathway Analysis of Differential Lipid Metabolites

**FIGURE 6:**
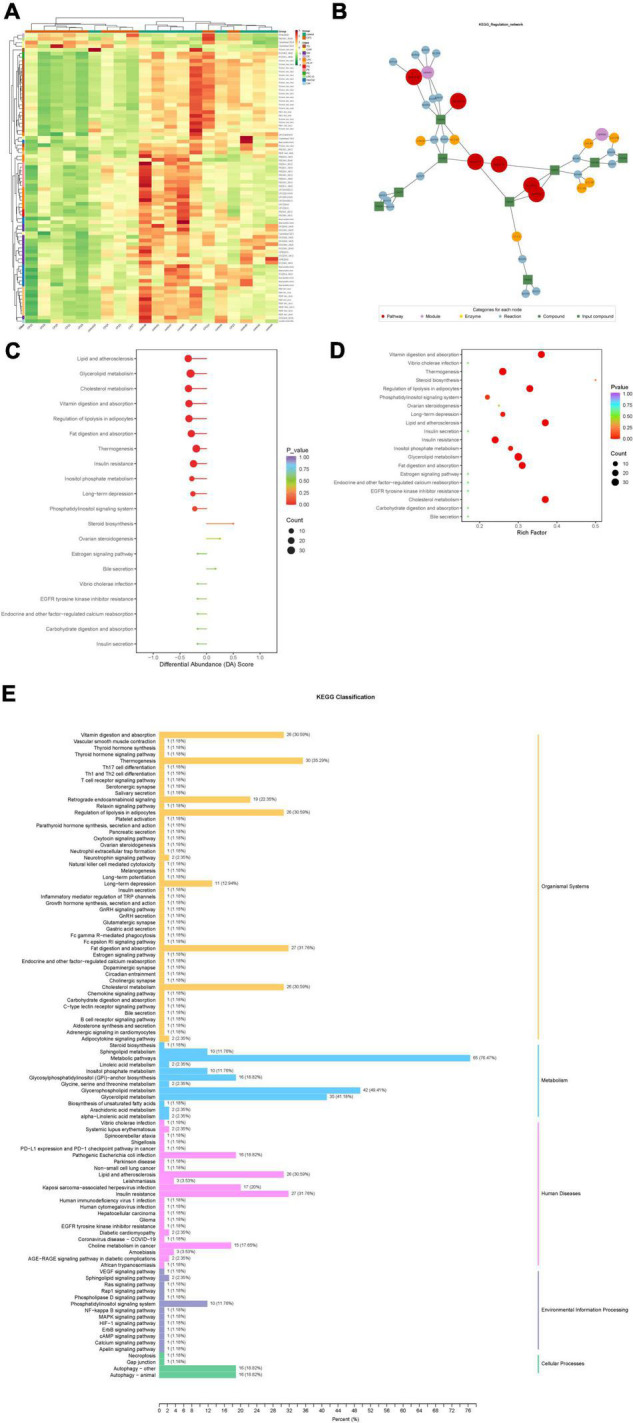
**(A)** Clustering analysis for different metabolites of KEGG signaling pathway. **(B)** Analysis of the regulatory network for differential metabolites. **(C)** Differential abundance score of differential metabolic pathways. **(D)** KEGG enrichment map of differential metabolites. **(E)** KEGG enrichment analysis of differentially significant lipid metabolites.

Based on the KEGG annotation information for the differential metabolites, KEGG metabolic pathways containing at least five differential metabolites were selected, and the contents of all differential metabolites in these pathways were clustered and analyzed to investigate better the patterns of changes in the contents of substances in potentially important metabolic pathways in different groupings (Figure 6A).

After obtaining the matching information of the differential metabolites, pathway search and regulatory interaction network analysis was performed based on the KEGG database of the corresponding species, and a network plot was presented in Figure 6B. In this network plot, the red dot represents a metabolic pathway, the yellow dot represents substance-related regulatory enzyme information, the green dot represents a background substance of a metabolic pathway, the purple dot represents a class of substance molecular module information, the blue dot represents a substance chemical interaction reaction, and the green square represents the difference substance obtained from this comparison.

The differential abundance score (DA Score) (Figure 6C) is a pathway-based analysis of metabolic changes, and the score captures the overall changes of all metabolites in a pathway. The DA score reflects the overall change of all metabolites in the metabolic pathway. A score of 1 indicates an up-regulated trend in the expression of all identified metabolites in the pathway and −1 a down-regulated trend in the expression of all identified metabolites in the pathway.

Based on the differential metabolite results, we constructed a KEGG enrichment map (Figure 6D) for differential metabolites, the rich factor is the ratio of the number of differentially expressed metabolites in the corresponding pathway to the total number of metabolites annotated by the pathway detection, and a more significant value indicates a greater enrichment. The *p*-value in the diagram is the hypergeometric test *p*-value. In the KEGG enrichment map of differential metabolites, the horizontal coordinate indicates the rich factor for each pathway, the vertical coordinate is the name of the pathway, and the color of the dot is the *p*-value. The size of the dots represents the number of differential metabolites enriched.

The total results of KEGG annotation of differentially significant metabolites were classified according to the type of pathway in KEGG, and the classification diagram is shown in Figure 6E.

### Transcriptomics Analysis

#### Quantitative Analysis of Gene Expression

**FIGURE 7:**
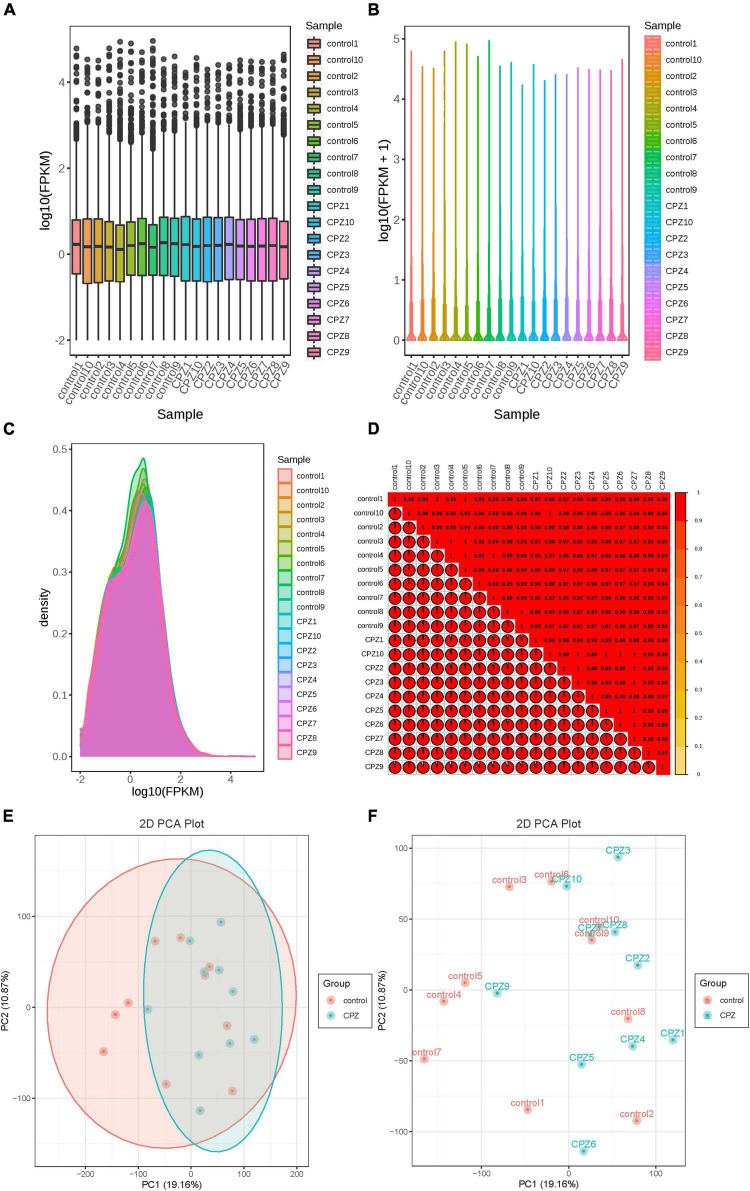
**(A)** Box line diagram. **(B)** Violin plot. **(C)** Density distribution graph. **(D)** correlation heatmap of R^2^. **(E,F)** The plots of PCA.

The fragments per kilobase of transcript per million fragments mapped (FPKM) was used to measure the transcript or gene expression level. The box line diagram (Figure 7A), violin plot (Figure 7B), and density distribution graph (Figure 7C) were used to display the dispersion, probability density, and concentrated interval of the gene expression level distribution for each sample, respectively. R (Pearson’s correlation coefficient) was used to assess the correlation of biological replicates of samples and the reliability of differentially expressed genes. The closer the R^2^ is to 1, the stronger the correlation between the two replicate samples. Our results showed that the R^2^ of the samples in this study were all higher than 0.8 (Figure 7D). The PCA analysis of the transcriptomics was shown in Figures 7E,F. The ellipse is the confidence interval, the horizontal coordinate is the first principal component (PC1), and the vertical coordinate is the second principal component (PC2).

#### Screen for Differential Genes

**FIGURE 8:**
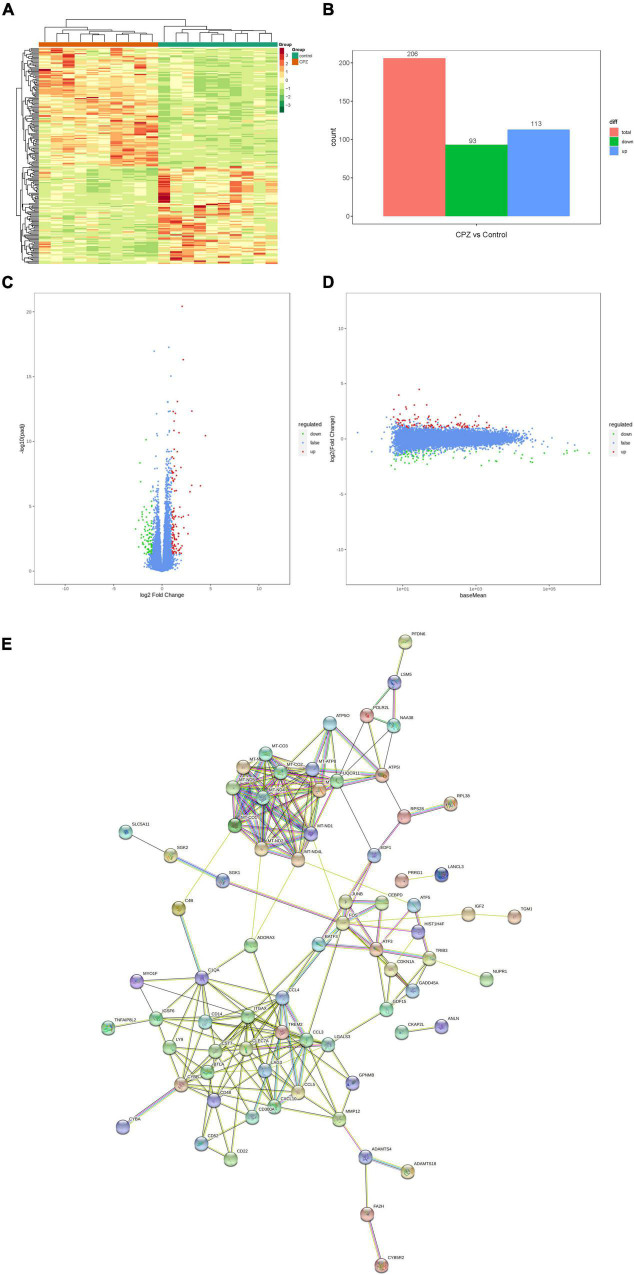
**(A)** Clustering heat map of FPKM. **(B)** Histogram of differential genes’ statistic. **(C,D)** Volcano map and M-vs.-A plot of all genes. **(E)** Differential genes protein interaction network.

In this study, the screening conditions for differential genes were log_2_Fold Change ≥ 1 and FDR < 0.05. The FPKM of differential genes’ centration and normalized expressions was used to map the clustering heat map of hierarchical cluster analysis; the clustering heat map (Figure 8A) showed a significant difference for the expression of genes between the control group and CPZ group. The results showed that 206 differential genes were screened, of which 113 were expressed up-regulated and 93 were expressed down-regulated (Figure 8B). In addition, the volcano map and M-vs.-A plot of all genes were displaced in Figures 8C,D. We used the STRING Protein Interaction Database (see text footnote 6) to map the differential gene protein interaction network (Figure 8E).

#### Enrichment Analysis of Difference Genes

**FIGURE 9:**
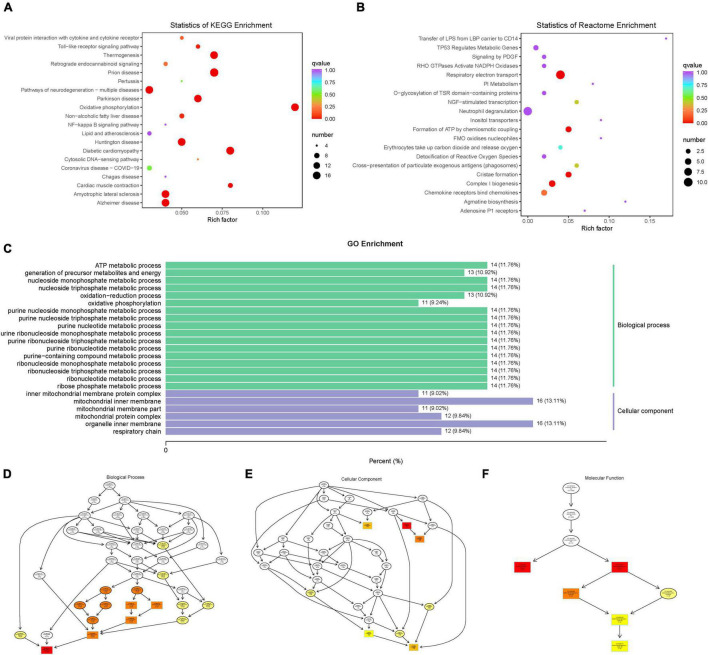
**(A)** scatter plot of KEGG enrichment analysis. **(B)** scatter plot of Reactome enrichment analysis. **(C)** histogram of GO enrichment analysis. **(D)** topGO directed acyclic graph of a biological process. **(E)** topGO directed acyclic graph of the cellular component. **(F)** topGO directed acyclic graph of molecular function.

Based on the KEGG enrichment analysis results, the top 20 pathways were selected to plot the scatter plot (Figure 9A). According to the Reactome enrichment analysis, the top 20 most significant pathways were chosen to draw a scatter plot (Figure 9B). In addition, we selected the most significant GO pathways from the GO enrichment results to plot the histogram (Figure 9C). Moreover, we make a topGO directed acyclic graph of the GO enriched term, including biological process (Figure 9D), cellular component (Figure 9E), and molecular function (Figure 9F). Explain here that the directed acyclic graph topGO visualizes differentially expressed gene enrichment GO nodes and their hierarchical relationships and is a graphical representation of the results of GO enrichment analysis of differentially expressed genes, with branches defining inclusion relationships and those from top to bottom, representing increasingly specific domains of functional description.

#### Integrated Analysis of Lipidomics and Transcript Profiles

**FIGURE 10:**
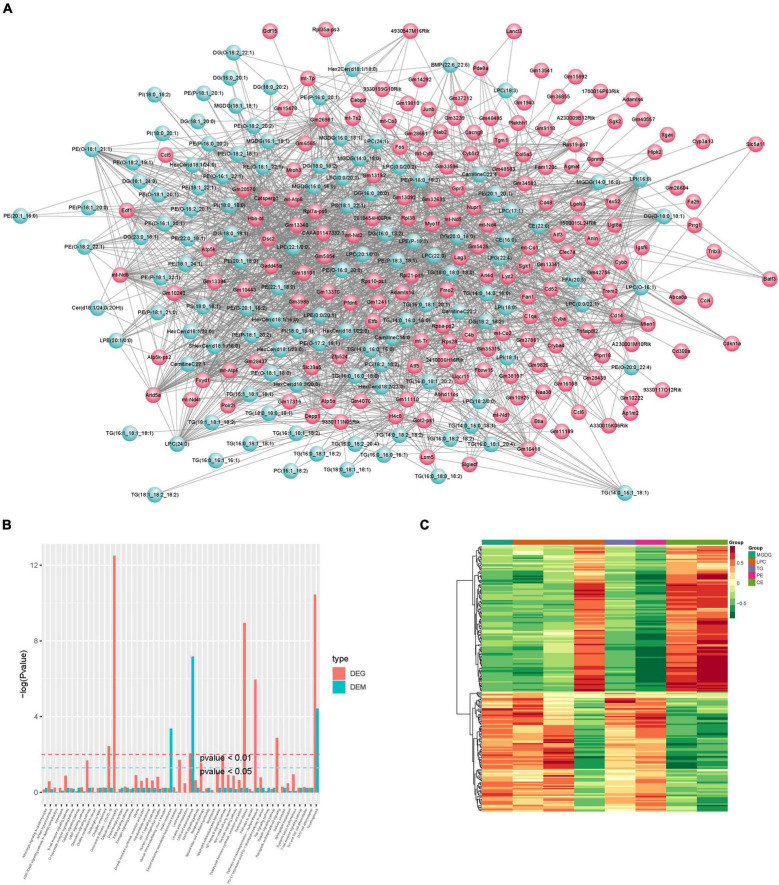
**(A)** Plot of total correlation network between differential metabolites and genes. **(B)** Histogram of pathways. **(C)** Heatmap of correlation coefficient clustering.

Correlation analysis was first performed for differential metabolites and genes detected between the control and CPZ groups. The core program of R software was used to calculate the Pearson correlation coefficients for differential metabolites and genes. According to the Pearson’s correlation coefficient (*r* > 0.6), the software of Cytoscape was used to plot the map of the total correlation network between differential metabolites and genes (Figure 10A); in addition, the above result was also displayed as the plot of the heatmap in Figure 10C. Based on the results of KEGG enrichment analysis for differential metabolites and genes, we drew a histogram of pathways (Figure 10B).

**FIGURE 11:**
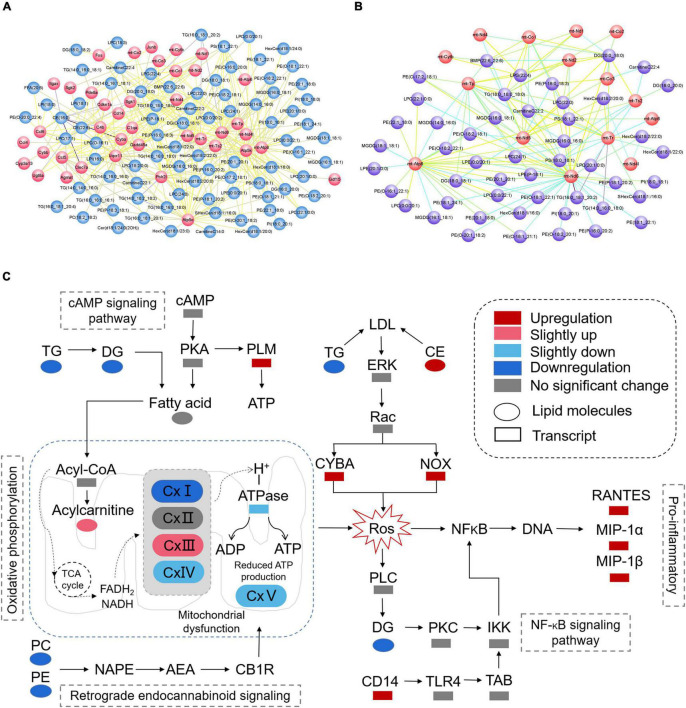
**(A)** Plot of oxidative stress correlation network. **(B)** Plot of mitochondrial function-related correlation network. **(C)** Pathway representation of mitochondrial dysfunction, oxidative stress, and neuroinflammation-related signaling pathways.

According to the results of KEGG enrichment analysis which was based on the differential metabolites and genes, we choose the MAPK signaling pathway, cAMP signaling pathway, NF-kappa B signaling pathway, HIF-1 signaling pathway, thermogenesis, retrograde endocannabinoid signaling, Parkinson’s disease, pathways of neurodegeneration—multiple diseases, lipid, and atherosclerosis which are associated to oxidative stress to plot the Figure 11A. In addition, the mitochondrial function-related genes and metabolites were used to draw Figure 11B. In summary, we extracted the above results to map Figure 11C to show the disorder’s biological processes of lipid metabolism and gene expression in CNS demyelinating diseases.

## Discussion

Lipid metabolism is a critical component of metabolomics since it is involved in energy transport, intercellular information exchange, and a variety of other biological activities. Targeted lipidomics research has recently emerged as a promising tool for elucidating the probable link between molecular pathways and disease patterns. To present, however, the mechanism through which lipid metabolism, mitochondrial dysfunction, oxidative stress, and neuroinflammation interact to cause the degenerative processes of CNS remyelination remains elusive. Thus, utilizing a self-built database with MRM detection mode, we performed qualitative and quantitative analysis of lipid metabolites in the corpus callosum of mice using the UPLC-MS/MS apparatus, creating detailed lipid profiles for a total of 867 lipids from 38 lipid classes. The KEGG enrichment analysis was performed first in the integrated analysis of lipidomics and transcript profiles. The results showed that the 120 significantly different lipid metabolites and 206 differential genes were mainly enriched in 51 KEGG pathways. By further analysis of the above enrichment pathways revealed that the MAPK signaling pathway, cAMP signaling pathway, NF-kappa B signaling pathway, HIF-1 signaling pathway, thermogenesis, retrograde endocannabinoid signaling, Parkinson’s disease, pathways of neurodegeneration—multiple diseases, lipid, and atherosclerosis are associated to oxidative stress. A deeper understanding of the complicated interaction between lipid metabolism, mitochondrial dysfunction, oxidative stress, and neuroinflammation in CNS demyelination may aid in the discovery of novel pharmaceutical targets for neuroprotective therapy. The differentially expressed genes and lipid metabolites from the above pathways related to oxidative stress were utilized to generate a map combining lipid metabolism and gene expression.

Mitochondria play an important role in oxidative phosphorylation, fatty acid oxidation, apoptosis, and calcium homeostasis (Witte et al., 2014). It also constantly generates reactive oxygen species (ROS), which can cause cell damage if the generation of ROS becomes excessive (Lin and Beal, 2006). Due to the brain’s high energy requirements, it is particularly susceptible to mitochondrial malfunction, which manifests itself in neurological illnesses (DiMauro et al., 2013). Its malfunction is a significant contributor to reversible neurological impairments in neuroinflammatory illnesses such as multiple sclerosis (Sadeghian et al., 2016). An energy imbalance appears to be a prominent component of the brain and spinal cord in MS (Vidaurre et al., 2014). Up to now, the mechanism of mitochondrial dysfunction in CNS demyelinating disorders has not yet been elucidated.

The majority of ATP synthesis is dependent on the system of interconnected ATP synthases (ComplexV) and the mitochondrial electron transport chain, which is composed of four enzymes and is housed within the inner mitochondrial membrane: reduced nicotinamide-de adenine dinucleotide (NADH) dehydrogenase (ComplexI), succinate dehydrogenase (ComplexII), cytochrome c oxidoreductase (ComplexIV) (Mancini et al., 2018). Reduced ComplexI activity, the principal electron entry site into the respiratory chain, is expected to result in a reduction in mitochondrial membrane potential, the rate at which electrons enter and exit the respiratory system (Nicholls, 2002). The results of transcriptomics indicated that the expression of mitochondrially encoded NADH dehydrogenase 1 (mt-Nd1), mitochondrially encoded NADH dehydrogenase 2 (mt-Nd2), mitochondrially encoded NADH dehydrogenase 4 (mt-Nd4), mitochondrially encoded NADH dehydrogenase 4L (mt-Nd4l), mitochondrially encoded NADH dehydrogenase 5 (mt-Nd5) and mitochondrially encoded NADH dehydrogenase 6 (mt-Nd6) were all down-regulated in the CPZ group. The results above demonstrate that ComplexI expression is down-regulated in the demyelinated corpus callosum. Sadeghian et al. (2016) discovered that EAE mice’s spinal cords have decreased ComplexI activity, but ComplexII activity remained unaltered. Additionally, our transcriptomics data revealed no significant difference in ComplexII levels between the control and CPZ groups. This may imply that ComplexII is relatively stable in the CPZ hypothesis.

In a previous investigation, ComplexIII activity was shown to be considerably increased in the spinal cords of EAE mice (Ng et al., 2019). Our data indicate that the ubiquinol-cytochrome c reductase2C complex III subunit XI (Uqcr11) was up-regulated in ComplexIII (log_2_FC: 1.229). On the other hand, cytochrome b (mt-Cytb) expression was decreased (log_2_FC: −1.026) in the CPZ group. According to the log_2_FC values for Uqcr11 and mt-Cytb, ComplexIII’s general trend was up-regulated. This could be to make up for mitochondrial enzymes that have been damaged or to keep the oxidative phosphorylation balance stable during the illness state. Complex IV is critical for mitochondrial function since it is the final component of the electron transport chain and accounts for about 90% of the total oxygen requirement of the cell (DiMauro and Schon, 2003). Additionally, Mahad et al. (2009) discovered that the content of ComplexIV and mitochondria in degenerated axons do not grow in a compensatory manner. Prior research established that exposure to EAE enhanced mitochondrial activity in demyelinated axons, but this increase was not accompanied by an increase in ComplexIV activity in their mitochondria. Additionally, ComplexIV activity was shown to be related to the extent of axonal damage but not to the quantity of mitochondrial material in the demyelinated lesion (Campbell and Mahad, 2018). In the EAE model, the decline in ComplexIV activity may start before there are changes in the structure of axonal mitochondria in the brain (Nikic et al., 2011). Except for cytochrome c oxidase subunit 8B (Cox8b), our results indicated that the expression of mitochondrially encoded cytochrome c oxidase III (mt-Co3), mitochondrially encoded cytochrome c oxidase I (mt-Co1), and mitochondrially encoded cytochrome c oxidase II (mt-Co2) was all decreased in the CPZ group. Thus, the general trend of ComplexIV expression was decreased in demyelinated corpus callosum tissue. Broadwater et al. (2011) used mass spectrometry to identify proteomic changes associated with mitochondrial respiratory chain complexes in the motor cortex of patients with multiple sclerosis and confirmed a significant decrease in the content of mitochondrial respiratory complexes ComplexIV and ComplexV via western blotting. Both mitochondrial ATP synthase 6 (mt-Atp6) (log_2_FC: −1.746) and mitochondrial ATP synthase 8 (mt-Atp8) (log_2_FC: −1.711) expression levels were decreased. On the other hand, Atp5o (log_2_FC: 1.048) and Atp5k (log_2_FC: 1.485) expression levels were increased. Thus, ComplexV’s overall tendency was finally down-regulated in the CPZ group.

Overproduction of ROS is intimately linked to caspase activation and cytochrome C release, both of which result in apoptosis (Ott et al., 2007). Oxidative stress caused by ROS can damage mitochondrial membrane lipids, enzyme complexes, and mitochondrial DNA, causing mitochondrial function to be harmed and causing less ATP to be made (Claus et al., 2013). Inflammation of the central nervous system (CNS) is a potent initiator of reactive oxygen species and mitochondrial dysfunction. As a result, limiting inflammation may be a beneficial strategy for avoiding mitochondrial dysfunction and increasing mitochondrial synthesis. Inflammation and neuro-axonal degeneration have been linked to MS, implying that immunological responses may exacerbate neurodegeneration, resulting in irreversible disease progression. This process may be affected by a lack of energy and problems with mitochondria caused by inflammation (Mancini et al., 2018). Furthermore, metabolic alterations may occur prior to demyelination or axonal degeneration (Sadeghian et al., 2016).

Phosphatidylcholine (PC) is the most abundant glycerophospholipid found in cell membranes and accounts for a significant portion of the composition of cell membranes, with phosphatidic acid and choline serving as the primary breakdown products. Phosphatidylethanolamine (PE) is the second most common phospholipid in the body, and it is found in cell and mitochondrial membranes. The results of targeted lipidomics show that both PC and PE concentrations were lowered in the CPZ group, which is consistent with earlier findings. The alteration of PE and PC demonstrated a dysregulation of glycerophospholipid metabolism in the demyelinated corpus callosum. Furthermore, we discovered a triglyceride metabolism abnormality in the demyelinated corpus callosum, with the content of TG and DG both decreasing while the level of cholesteryl ester (CE) was increasing.

Based on the integrated analysis of targeted lipidomics and transcriptomics (Figures 10, 11), the following hypothesis is advanced: by interfering with mitochondrial activity, lower levels of PE and PC, as well as lower levels of TG and DG, in the demyelinated corpus callosum may result in energy depletion and an increase in ROS. In addition, high CE levels and low TG levels may lead to more Cytochrome b-245, alpha polypeptide (CYBA) and NADPH oxidase 2 (NOX) expression in the demyelinated corpus callosum. Interestingly, CYBA and NOX may contribute to an increase in ROS. Finally, higher ROS levels in the body may result in increased expression of pro-inflammatory chemicals such as C-C motif chemokine 5 (RANTES), C-C motif chemokine 3 (MIP-1α), and Ccl4, chemokine (C-C motif) ligand 4 (MIP-1β).

In summary, the beginning and progression of CNS demyelination is a complicated pathological process that may be mediated by a combination of lipidomics and transcriptomics. A single omics analysis may not be able to identify the overall characteristics of demyelination or provide a precise mechanism for how this process takes place completely. By using targeted lipidomics and transcriptomics, we were able to reveal the potential crosstalk between disorders of lipid metabolism, mitochondrial dysfunction, oxidative stress, and neuroinflammation in the corpus callosum of the CPZ model. The results described above may serve as a blueprint for the identification of effective therapeutic targets for the treatment of CNS demyelinating disorders.

## Data Availability Statement

The datasets presented in this study can be found in online repositories. The names of the repository/repositories and accession number(s) can be found below: NCBI (accession: PRJNA816699).

## Ethics Statement

The animal study was reviewed and approved by the Laboratory Animal Ethical and Welfare Committee Tong Ren Hospital affiliated to Shanghai Jiao Tong University School of Medicine (AF/SC-11/02.1).

## Author Contributions

Z-JZ, R-ZZ, X-JW, and X-YL conceived and designed the experiments. Z-JZ, R-ZZ, and C-YZ performed the experiments. C-YZ, X-HD, and T-QL collected the data. X-YL and X-HD contributed the reagents, materials, and analysis tools. Z-JZ and X-JW analyzed the data and wrote the article. X-HD conducted quality control on the articles and guided the submission. All authors read and approved the final manuscript.

## Conflict of Interest

The authors declare that the research was conducted in the absence of any commercial or financial relationships that could be construed as a potential conflict of interest. The reviewer JT declared a shared affiliation with the author T-QL to the handling editor at the time of review.

## Publisher’s Note

All claims expressed in this article are solely those of the authors and do not necessarily represent those of their affiliated organizations, or those of the publisher, the editors and the reviewers. Any product that may be evaluated in this article, or claim that may be made by its manufacturer, is not guaranteed or endorsed by the publisher.

## Footnotes

1
https://sciex.com.cn/

2
https://sciex.com

3
www.r-project.org

4
http://www.kegg.jp/kegg/compound/

5
http://www.kegg.jp/kegg/pathway.html

6
http://string-db.org

## Abbreviations

CNS: Central nervous system
MS: Multiple sclerosis
CPZ: Cuprizone
QC: Quality control
SD: Standard deviation
CV: Coefficient of Variation
ANOVA: One-way analysis of variance
VIP: Variable influence on projection
TIC: Total ion flow chromatogram
PCA: Principal component analysis
OPLS-DA: Orthogonal Partial least squares discriminant analysis
PCC: Pearson correlation coefficients
HCA: Hierarchical cluster analysis
DA Score: differential abundance score
PE: Phosphatidylethanolamine
PC: Phosphatidylcholine
DAG: Diacylglycerol
CE: Cholesteryl ester
ROS: Reactive oxygen species
CD14: Monocyte differentiation antigen
MIP-1 β: Ccl4 chemokine (C-C motif) ligand 4
MIP-1 α: C-C motif chemokine 3
NOX: NADPH oxidase 2
CYBA: Cytochrome b-245 alpha polypeptide
RANTES: C-C motif chemokine 5
mt-Nd1: Mitochondrially encoded NADH dehydrogenase 1
mt-Nd2: Mitochondrially encoded NADH dehydrogenase 2
mt-Nd4: Mitochondrially encoded NADH dehydrogenase 4
mt-Nd4l: Mitochondrially encoded NADH dehydrogenase 4L
mt-Nd5: Mitochondrially encoded NADH dehydrogenase 5
mt-Nd6: Mitochondrially encoded NADH dehydrogenase 6
Uqcr11: Ubiquinol-cytochrome c reductase%2C complex III subunit XI
mt-Cytb: Mitochondrially encoded cytochrome b
mt-Co3: Mitochondrially encoded cytochrome c oxidase III
mt-Co1: Mitochondrially encoded cytochrome c oxidase I
mt-Co2: Mitochondrially encoded cytochrome c oxidase II
Cox8b: Cytochrome c oxidase subunit 8B
mt-Atp6: Mitochondrially encoded ATP synthase 6
mt-Atp8: Mitochondrially encoded ATP synthase 8.
